# Assessing ChatGPT’s theoretical knowledge and prescriptive accuracy in bacterial infections: a comparative study with infectious diseases residents and specialists

**DOI:** 10.1007/s15010-024-02350-6

**Published:** 2024-07-12

**Authors:** Andrea De Vito, Nicholas Geremia, Andrea Marino, Davide Fiore Bavaro, Giorgia Caruana, Marianna Meschiari, Agnese Colpani, Maria Mazzitelli, Vincenzo Scaglione, Emmanuele Venanzi Rullo, Vito Fiore, Marco Fois, Edoardo Campanella, Eugenia Pistarà, Matteo Faltoni, Giuseppe Nunnari, Annamaria Cattelan, Cristina Mussini, Michele Bartoletti, Luigi Angelo Vaira, Giordano Madeddu

**Affiliations:** 1 Unit of Infectious Diseases, Department of Medicine, Surgery, and Pharmacy, University of Sassari, Sassari, Italy; 2 Unit of Infectious Diseases, Department of Clinical Medicine, Ospedale dell’Angelo, Venice, Italy; 3 Unit of Infectious Diseases, Department of Clinical Medicine, Ospedale Civile S.S. Giovanni e Paolo, Venice, Italy; 4 Unit of Infectious Diseases, Department of Clinical and Experimental Medicine, ARNAS Garibaldi Hospital, University of Catania, Catania, Italy; 5 Infectious Diseases Unit - IRCCS Humanitas Research Hospital, Via Manzoni 56, Rozzano, Milan, 20089 Italy; 6 Department of Biomedical Sciences, Humanitas University, Via Rita Levi Montalcini 4, Pieve Emanuele, Milan, 20090 Italy; 7 Infectious Diseases Service, Cantonal Hospital of Sion and Institut Central des Hôpitaux (ICH), Sion, Switzerland; 8 Institute of Microbiology, Department of Laboratory Medicine and Pathology, Lausanne University Hospital, Lausanne, Switzerland; 9 University of Modena and Reggio Emilia, Modena, Italy; 10 Infectious and Tropical Diseases Unit, Padua University Hospital, Padua, Italy; 11 Unit of Infectious Diseases, Department of Clinical and Experimental Medicine, University of Messina, Messina, Italy; 12 Maxillofacial Surgery Unit, Department of Medicine, Surgery, and Pharmacy, University of Sassari, Sassari, Italy; 13 PhD School in Biomedical Science, Biomedical Science Department, University of Sassari, Sassari, Italy

**Keywords:** ChatGPT, Bacterial infections, Artificial intelligence, Endocarditis, Abdominal infection, Pneumonia, Blood-stream infection, Infectious diseases, Antibiotic resistance, Antimicrobial stewardship

## Abstract

**Objectives:**

Advancements in Artificial Intelligence(AI) have made platforms like ChatGPT increasingly relevant in medicine. This study assesses ChatGPT’s utility in addressing bacterial infection-related questions and antibiogram-based clinical cases.

**Methods:**

This study involved a collaborative effort involving infectious disease (ID) specialists and residents. A group of experts formulated six true/false, six open-ended questions, and six clinical cases with antibiograms for four types of infections (endocarditis, pneumonia, intra-abdominal infections, and bloodstream infection) for a total of 96 questions. The questions were submitted to four senior residents and four specialists in ID and inputted into ChatGPT-4 and a trained version of ChatGPT-4. A total of 720 responses were obtained and reviewed by a blinded panel of experts in antibiotic treatments. They evaluated the responses for accuracy and completeness, the ability to identify correct resistance mechanisms from antibiograms, and the appropriateness of antibiotics prescriptions.

**Results:**

No significant difference was noted among the four groups for true/false questions, with approximately 70% correct answers. The trained ChatGPT-4 and ChatGPT-4 offered more accurate and complete answers to the open-ended questions than both the residents and specialists. Regarding the clinical case, we observed a lower accuracy from ChatGPT-4 to recognize the correct resistance mechanism. ChatGPT-4 tended not to prescribe newer antibiotics like cefiderocol or imipenem/cilastatin/relebactam, favoring less recommended options like colistin. Both trained- ChatGPT-4 and ChatGPT-4 recommended longer than necessary treatment periods (p-value = 0.022).

**Conclusions:**

This study highlights ChatGPT’s capabilities and limitations in medical decision-making, specifically regarding bacterial infections and antibiogram analysis. While ChatGPT demonstrated proficiency in answering theoretical questions, it did not consistently align with expert decisions in clinical case management. Despite these limitations, the potential of ChatGPT as a supportive tool in ID education and preliminary analysis is evident. However, it should not replace expert consultation, especially in complex clinical decision-making.

**Supplementary Information:**

The online version contains supplementary material available at 10.1007/s15010-024-02350-6.

## Introduction

In the last year, researchers have focused on using ChatGPT in medicine and education [1, 2]. ChatGPT has rapidly emerged as a significant asset in medicine, facilitating a range of applications from diagnostic support to patient education and administrative tasks [3, 4]. Its ability to quickly process and interpret vast amounts of medical literature and patient data makes it a valuable tool for suggesting diagnoses and potential treatment plans, enhancing the efficiency and accuracy of medical practices [5–7]. Regarding medical education, ChatGPT acts as an interactive tool, enabling both students and seasoned professionals to refine their clinical skills through simulated patient interactions [1, 8]. Ayers et al. demonstrated that evaluators preferred ChatGPT’s responses to patient questions over those of physicians, highlighting its ability to provide quality and empathetic answers [9]. Additionally, it streamlines administrative processes by automating the documentation of patient histories and generating comprehensive discharge summaries, freeing up valuable time for healthcare providers to focus on patient care. However, integrating ChatGPT into healthcare systems also brings forth critical ethical considerations [3], particularly concerning patient confidentiality and the reliability of digitally generated advice. To ensure the safe deployment of such tools in clinical settings, rigorous validation and adherence to strict privacy regulations are imperative, underscoring the need for a balanced approach that harnesses the benefits of chatbots while mitigating potential risks.

Recent literature reveals disparities in practice between AI systems like ChatGPT and human specialists, especially in complex decision-making areas. Research, like those conducted by Al Tibi et al., has demonstrated a significant difference in the recommended course of treatment for hypertension between ChatGPT and cardiologist [10]. Furthermore, Massey et al. demonstrated that ChatGPT significantly underperformed orthopaedic residents when asked to conduct an orthopaedic assessment examination [11]. These studies demonstrate how ChatGPT frequently fails to comprehend intricate clinical scenarios.This could be particularly evident in specialties such as Infectious Diseases, where the interpretation of dynamic and complex clinical data is crucial. The use of ChatGPT in Infectious Diseases has been investigated by different authors, focusing on specific domains. Recent discussions, such as the correspondence by Howard et al. in Lancet Infectious Diseases, have highlighted both the potential and limitations of using ChatGPT in clinical settings, particularly in providing antimicrobial advice. This has sparked significant debate regarding the role of AI in augmenting or potentially replacing traditional roles in infectious disease management [12–15]. However, in this work, the same people created the cases, asked the question, and evaluated the answer. In our study, we aim to explore the application of ChatGPT in providing diagnostic insights and treatment recommendations based on antibiograms, which are critical tools in identifying antibiotic susceptibilities and comparing them to medical residents and specialists in Infectious Diseases.

## Methods

### Study design and participants

We conducted a comparative study to assess the ability of ChatGPT4 to reply to medical questions about Infectious Diseases. To do so, three specialists in Infectious Diseases (A.D.V., N.G., G.M.) formulated 72 queries focused on four different topics: endocarditis, bloodstream infection (BSI), pneumonia, and intra-abdominal infections (IAI). Each topic included six true or false questions, six open-ended questions, and six clinical cases with antibiograms. The questions have been created using different difficulty levels; in particular, two easy, two medium, and two hard questions were formulated. The list of questions is available in Table S1.

### Data collection

The 72 questions were administered to the eight participants and ChatGPT, including four residents in the last year of Infectious Diseases and four specialists with over three years of experience but less than ten. Participants were allowed to use any necessary resources to answer the questions except ChatGPT or similar tools. For ChatGPT, queries were entered manually, and responses were directly collected from the interface. The prompt used in ChatGPT is available in Supplemental material (S2). The process was identical for both the standard and trained versions of ChatGPT-4, facilitating comparative analysis of the enhancements training on specialized datasets provided. The trained version of ChatGPT-4 was developed using the GPT-builder tool, incorporating international guidelines, randomized clinical trials, systematic reviews, and meta-analyses related to the four topics. We used only open-access articles to avoid copyright issues. The list of papers and guidelines is reported in supplemental materials.

### Blind review and evaluation

All responses were anonymized and reviewed by a blinded panel of experts who have been published several manuscripts about bacterial infections and antibiotic treatments. The panel assessed the responses for accuracy and completeness. The true or false questions were evaluated as correct and not correct. Accuracy was evaluated using a six-point Likert scale, where (1) represented a completely incorrect response; (2) indicated the presence of more incorrect than correct elements; (3) suggested an equal balance of correct and incorrect elements; (4) denoted the presence of more correct than incorrect elements; (5) was used for an almost fully correct response; and 6 for an entirely correct response. Completeness was assessed using a three-point Likert scale: (1) stood for an incomplete answer that addressed only some aspects of the question with significant parts missing; (2) represented an adequate answer covering all necessary aspects of the question; and (3) denoted a comprehensive response that covered all aspects of the question and offered additional information or context beyond expectations.

For the clinical scenarios and antibiograms, the panel evaluated:

i) the ability of participants and ChatGPT to identify the resistance mechanism based on the phenotype of bacteria present in the antibiograms, where responses were classified as (1) completely wrong, (2) partially correct, or (3) correct;
ii) the appropriateness of the prescribed antibiotics (type and dosage), with feedback categorized as (1) completely wrong, (2) partially correct, (3) correct, or (4) overtreatment;
iii) the adequacy of treatment duration, assessed as (1) too short, (2) adequate, or (3) too long.

### Statistical analysis

Statistical methods were employed to compare the performance across different respondent groups (residents, specialists, standard ChatGPT-4, and trained ChatGPT-4). Data have been described using absolute numbers and percentages. Accuracy has also been described using median and interquartile range (IQR). Chi-squared test was used to assess the presence of differences between groups. We also evaluate differences in accuracy between groups using the Kruskal-Wallis test. Statistical significance was set at p-values of less than 0.05, and data analysis was carried out through STATA (Version 16.1 StataCorp, College Station, TX, USA).

### Ethical considerations

Given the nature of the study involving only de-identified, hypothetical clinical scenarios and no real patient data, ethical review exemption was sought and granted, aligning with the institutional guidelines on human subject research.

## Results

Overall, 720 responses were obtained and reviewed by a blinded panel of experts in antibiotic treatments.

### True or false

**Fig. 1:**
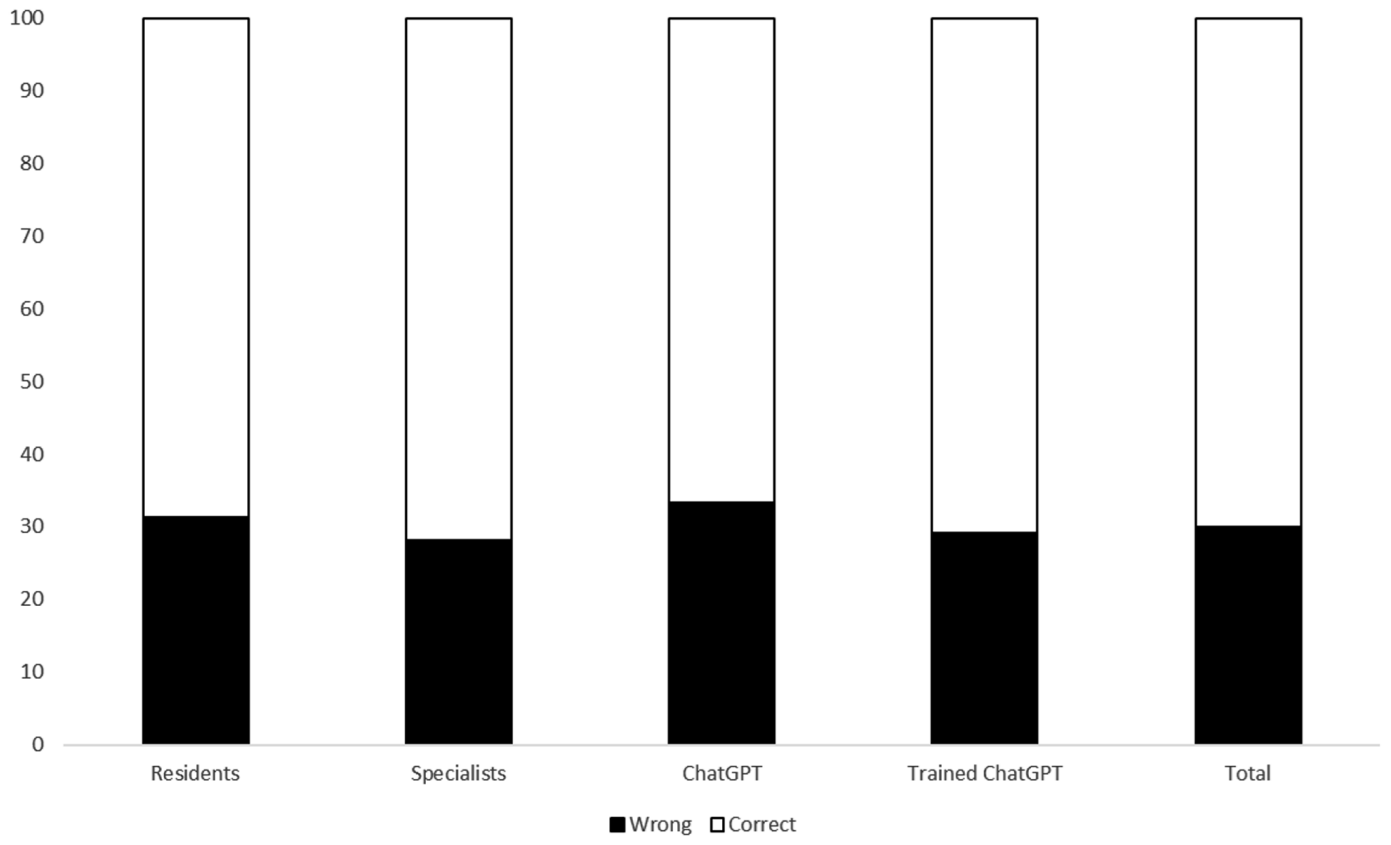
Percentage of correct and wrong answers for the true or false questions in the different groups

Among the four groups, no significant differences were noted for the true/false questions, with approximately 70% of the responses being correct across all groups (Fig. 1). Similar performance for easier and medium-difficulty questions was registered (Table S1). However, for the more challenging questions, the percentage of correct answers dropped; notably, ChatGPT-4 lacked in providing the correct solutions compared to the specialists, achieving only 37.5% accuracy versus 68.7%.

Regarding specific topics, a generally low percentage of correct answers was observed for pneumonia, with only 50% of corrected answers (Table S2). Interestingly, both the standard and trained versions of ChatGPT-4 outperformed human participants in responding to questions about endocarditis (83.3% vs. 70.8%). In contrast, human participants performed better on questions related to intra-abdominal infections (Figure S1).

### Open-ended questions

**Fig. 2:**
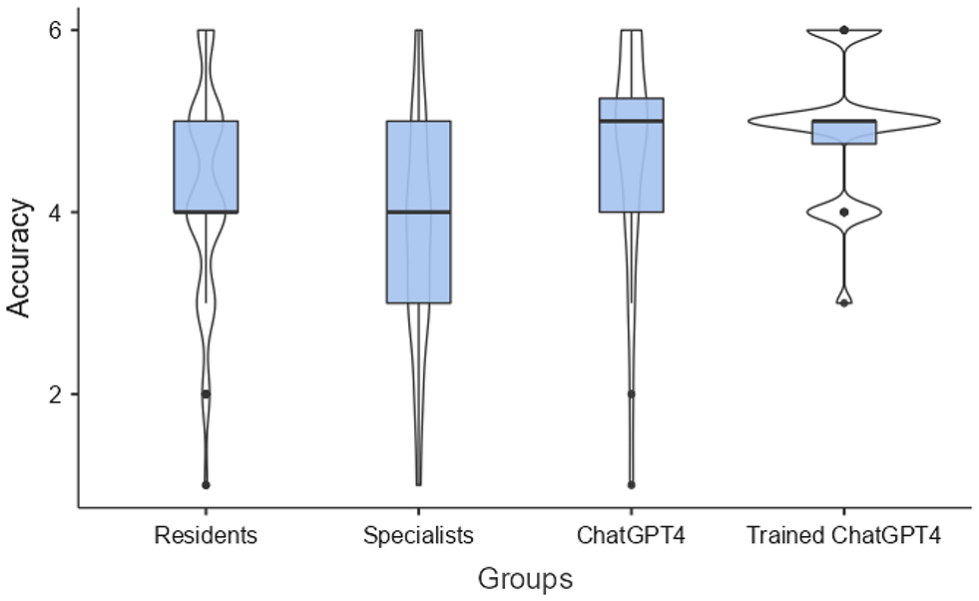
Performance of Infectious Diseases residents and specialists, ChatGPT4 and trained ChatGPT4 in answering open questions regarding antibiotic treatment. *p-*value calculated with Kruskal-Wallis test < 0.001

Regarding the open-ended questions, the trained ChatGPT-4 and standard ChatGPT-4 provided more accurate answers than both the residents and specialists (Fig. 2). The two ChatGPT-4 delivered more 5 and 6-point answers. In addition, trained ChatGPT4 received only one and ChatGPT4 only four scored below 4 points (Table 1).

**Table 1:** Accuracy scoring of infectious diseases residents, specialists, ChatGPT4, and trained ChatGPT4 in answering open-ended antibiotic questions (*p* = 0.004)

Accuracy	Residents	Specialists	ChatGPT4	Trained-ChatGPT4	Total
1	2 (2.1)	5 (5.2)	1 (4.1)	0	8 (3.4)
2	6 (6.3)	10 (10.4)	1 (4.1)	0	17 (7.1)
3	15 (15.6)	26 (27.1)	2 (8.4)	1 (4.1)	44 (18.3)
4	32 (33.3)	30 (31.2)	4 (16.7)	5 (20.8)	71 (29.6)
5	28 (29.2)	18 (8.8)	10 (41.7)	13 (54.1)	69 (28.7)
6	13 (13.5)	7 (7.3)	6 (25.0)	5 (20.8)	31 (12.9)

Trained ChatGPT also led in completeness, with a higher percentage of 3-point responses and no 1-point responses. ChatGPT-4 performed also better than residents and specialists (Table 2).

**Table 2:** Completeness scoring of infectious diseases residents, specialists, ChatGPT4, and trained ChatGPT4 in answering open-ended questions regarding antibiotics (*p*-value < 0.001)

Completeness	Residents	Specialists	ChatGPT4	Trained-ChatGPT4	Total
1	16 (14.6)	36 (37.5)	1 (4.2)	0	51 (21.2)
2	62 (64.6)	52 (54.2)	11 (45.8)	11 (45.8)	136 (56.7)
3	20 (20.8)	8 (8.3)	12 (50.0)	13 (54.2)	53 (22.1)

For both accuracy and completeness, no differences were observed across different difficulty levels (Tables S3-4). Regarding different topics, we have found that pneumonia-related questions received fewer 5 and 6-point responses for accuracy and 3-point responses for completeness compared to the other topics (Tables S5-6).

### Clinical cases

Regarding the clinical cases, we observed a lower accuracy of ChatGPT-4 in recognizing the correct resistance mechanisms, while the trained version of ChatGPT and human experts showed similar performances (Table 3).

**Table 3:** Performance of infectious diseases residents, specialists, ChatGPT4, and trained ChatGPT4 in identifying the correct resistance mechanisms according to the antibiogram (p-value 0.053)

Resistance Mechanism	Residents	Specialists	ChatGPT4	Trained-ChatGPT4	Total
Wrong	17 (17.7)	16 (16.67)	11 (45.8)	5 (20.8)	51 (21.2)
Partially correct	52 (54.2)	54 (56.2)	11 (45.8)	14 (58.4)	136 (56.7)
Correct	27 (28.1)	26 (27.1)	2 (8.4)	5 (20.8)	53 (22.1)

**Fig. 3:**
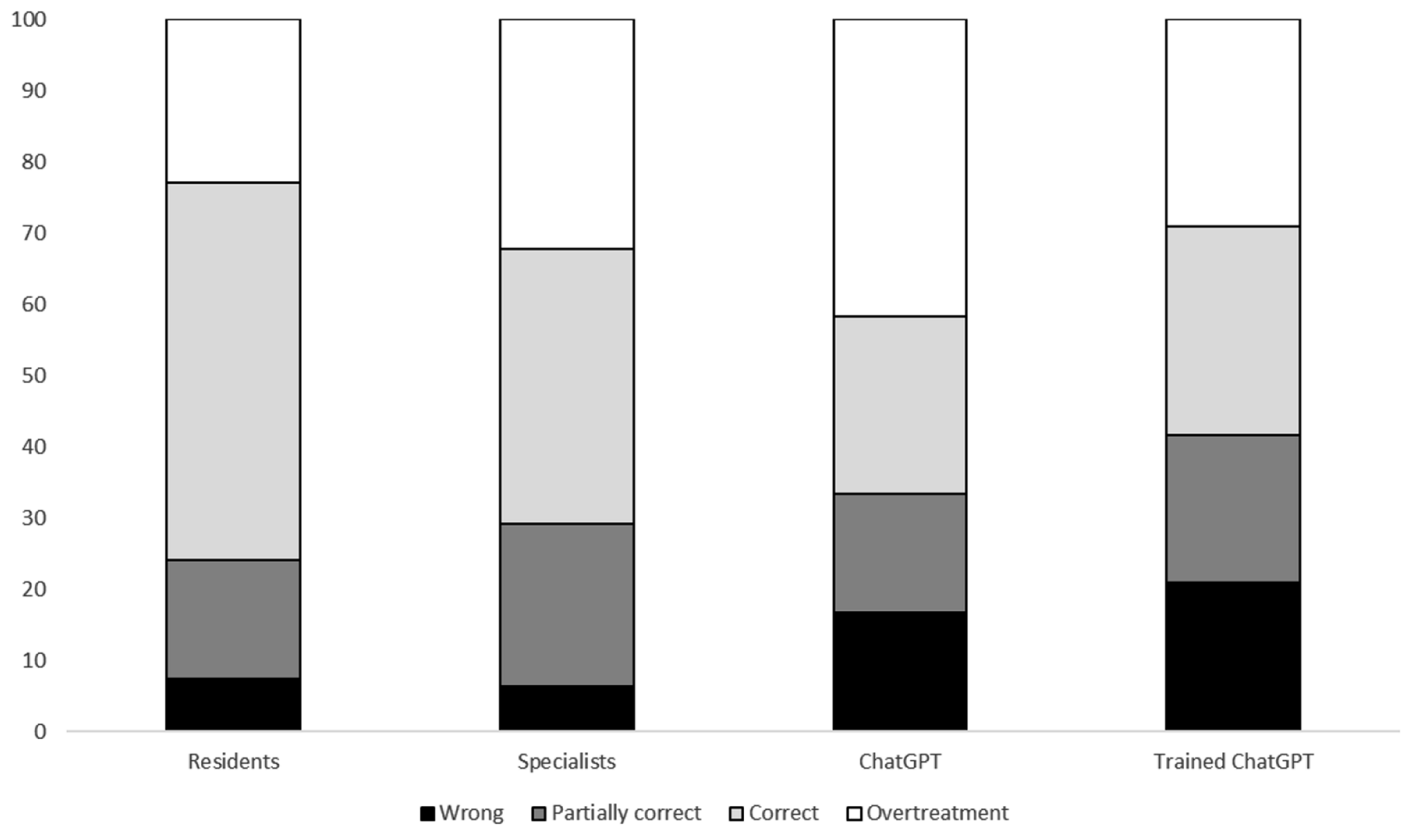
Performance of Infectious Diseases residents, specialists, ChatGPT4, and trained ChatGPT4 in prescribing the correct antibiotic treatment according to the clinical cases and the antibiograms. *p-*value 0.068

In choosing antibiotic treatments, ChatGPT-4 versions produced a higher rate of incorrect responses, and overtreatment with a borderline not-significative difference (*p* = 0.068) (Fig. 3). In particular, both standard and trained ChatGPT-4 tended not to prescribe newer antibiotics like cefiderocol or imipenem/cilastatin/relebactam, favoring less recommended options like colistin.

**Fig. 4:**
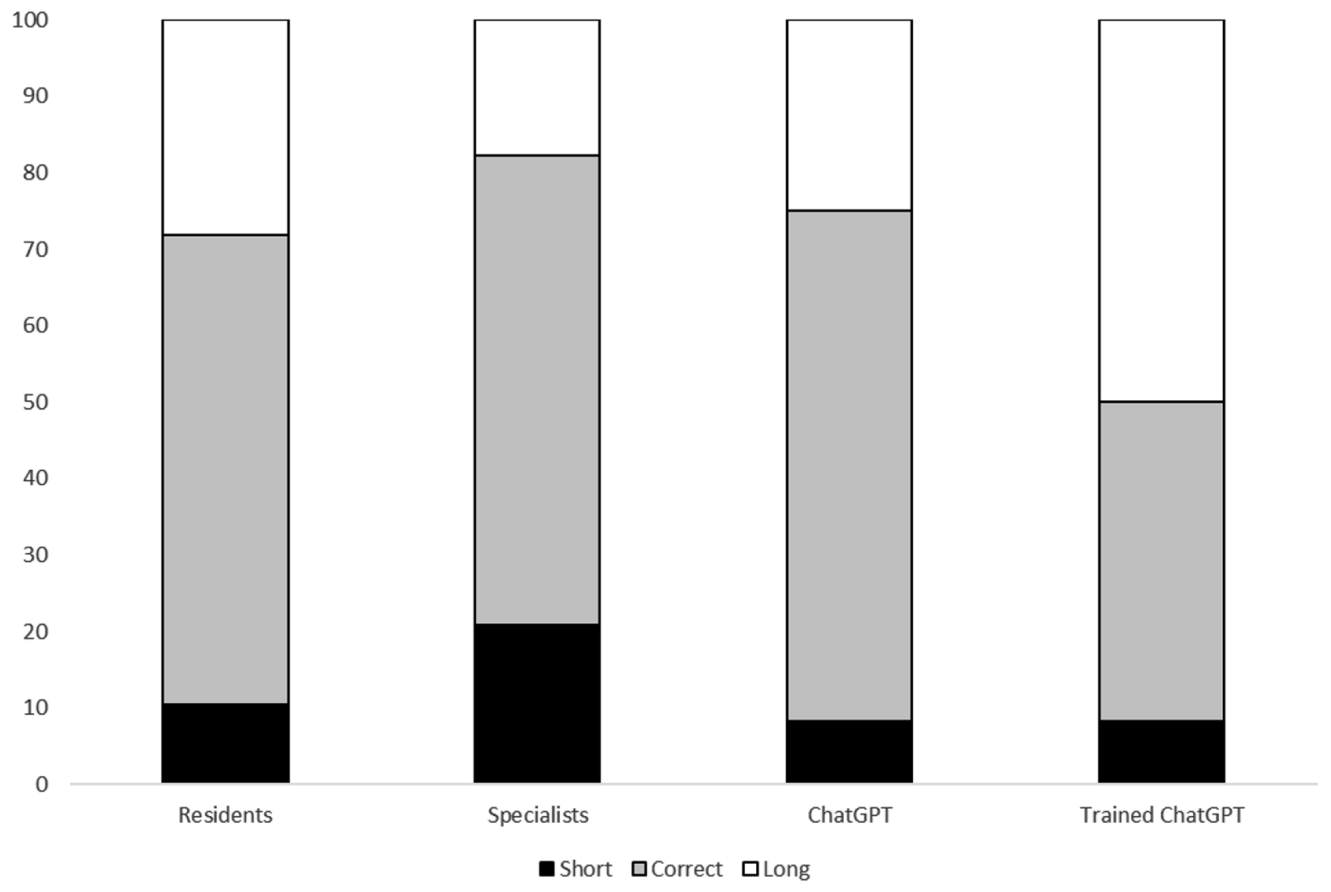
Performance of Infectious Diseases residents, specialists, ChatGPT4, and trained ChatGPT4 in prescribing the correct length of treatment according to the clinical cases. *p*-value = 0.022

Finally, trained ChatGPT-4 had a conservative approach regarding the treatment length, recommending longer than necessary treatment periods (Fig. 4).

The difficulty of the questions influenced resistance mechanism identification and antibiotic choice but not the duration of treatment. In particular, only 8/80 (10%) answers were correct for the more difficult questions. Regarding antibiotic prescriptions, we observed a high percentage of wrong and partially correct prescriptions for more difficult questions (Tables S7-9).

Focusing on the topics, we observed that pneumonia had the highest percentage of correct answers regarding the resistance mechanism, while endocarditis had the lowest percentage of wrong answers (Table S10). Pneumonia had the highest percentage of overtreatment, while BSI infection had the lowest percentage of overtreatment and the highest of correct answers (Table S11). Finally, about the length of treatment, the abdominal infection had the highest percentage of too long treatments, while endocarditis received the highest percentage of correct feedback (Table S12).

## Discussion

Our study assesses the capabilities of ChatGPT compared to ID residents and specialists, revealing the strengths and limitations of this advanced tool. The results indicate that ChatGPT performs well in answering true or false questions and open-ended queries. This efficiency stems from its ability to process and generate language based on the extensive data it has been trained on. However, it’s important to note that this does not equate to genuine understanding or reasoning, which are essential elements of human intelligence [16]. For this reason, the chatbot’s ability to deal with real-life scenarios is still to be questioned.

In more complex clinical scenarios, such as interpreting antibiograms, ChatGPT’s performance varies. The standard version of ChatGPT struggles in such context, whereas a trained version shows improved results, though it still does not fully match human clinicians’ nuanced judgment and experiential learning. This highlights the tool’s limitations in contexts that require deep understanding and the ability to integrate multiple data sources.

In addition, ChatGPT frequently recommended older antibiotic options such as colistin over newer treatments like cefiderocol or imipenem/cilastatin/relebactam. This tendency points to limitations in its programming and access to the most current medical guidelines, as well as likely to the higher number of available publications using older antibiotic regimens with a longer duration of treatments. Furthermore, ChatGPT does not account for the different national guidelines and varying accessibility to drugs across different parts of the world, particularly in countries with limited access to newer treatments.

Howard et al., in 2023, investigated the ability of ChatGPT to resolve clinical scenarios. They discussed eight clinical cases with the chatbot, asking for advice about the correct management [17]. Their study differs considerably from our design and research idea. In addition, when this study was conducted, ChatGPT could not navigate the internet and had to rely only on training. However, in their study, ChatGPT also failed to prescribe new antibiotics such as cefiderocol or ceftazidime/avibactam, proposing treatment with colistin and tigecycline.

Another interesting study was conducted by Mailliard et al., in which they prospectively submitted to ChatGPT4 data from 44 clinical cases, and these were managed by expert infectious diseases physicians. Two further experts compared the performance of the ChatGPT tool and the human colleagues. Overall, plans from ChatGPT were considered optimal in one case, satisfactory in 17, and harmful in 7 patients. These data confirm what we also suggest: ChatGPT is a promising and resourceful tool, but it cannot replace human medical decisions and should not be used recklessly [12]. Finally, also Tunçer and Güçlü investigated the accuracy of ChatGPT in answering questions about infectious diseases. In particular, they investigated several ID topics, including HIV, hepatitis, and bacterial infection, for 200 questions. They found an accuracy between 72.3% of correct answers for urinary tract infections and 90% for tuberculosis. The only topic in common with our study was pneumonia, where ChatGPT answered completely correctly in 77.3% of cases and partially correctly in 9% of cases. In 13.7% of cases, the answers were mixed or misleading [18].

This study has several limitations. Firstly, the difficulty of the questions was subjectively assessed by the experts who created them, which may not ensure a balanced representation across the different themes. Furthermore, we evaluated only four types of bacterial infection, so our results cannot be generalized for all bacterial infections or other infectious diseases. Additionally, the difficulty level was set based on the knowledge of the experts; this could explain why both residents and specialists made mistakes even on questions considered easier. Secondly, the study was conducted in English. Conducting the same study in other languages might yield different results, but we cannot predict the specific impact of the language choice.

Additionally, the sample size and scope were limited, restricting the generalizability of the findings to other medical specialities or broader clinical applications. The study’s reliance on hypothetical clinical scenarios without real patient interactions may not fully capture the complexities and nuances of actual clinical practice. Furthermore, the performance of ChatGPT could be influenced by the specific version used, as ongoing updates and training modifications could alter its effectiveness. Ethical and privacy considerations, particularly concerning data misuse and algorithmic biases, are critical and were not extensively explored in this study.

Finally, this work lacks real-world validation. A few studies have been published involving a real-life application of AI in clinical decision-making processes for infectious disease issues, with promising results; however, most studies have focused on applying machine learning algorithms [19, 20].

This study highlights that ChatGPT could be useful in medical education and as a preliminary diagnostic tool, providing initial advice or supplementing medical professionals’ knowledge. However, it is essential to remember that it is impossible, now and ever, to depend only on ChatGPT for clinical decisions. To understand the reasons, it is mandatory to remember how ChatGPT works. The system generates responses by analyzing patterns in the data it has been exposed to during training without any true understanding of the content. This method, based purely on statistical correlations, can result in inaccuracies, especially in complex medical scenarios. In addition, ChatGPT cannot perform physical examinations, detect non-verbal cues, or understand context as human doctors can. It also relies on the input it receives, and incorrect or incomplete information can lead to inaccurate recommendations [21]. This underscores the fact that ChatGPT should support, not replace, the judgment of healthcare professionals.

However, in regions with limited access to specialist consultations, ChatGPT could offer preliminary support, but it should not substitute professional judgment. The potential for using such tools in healthcare is significant, yet integrating them into routine clinical practice demands addressing substantial challenges, including ensuring accuracy, reliability, and patient privacy.

As development progresses, it is crucial to closely align these systems with clinical needs and ethical standards before they are considered viable for widespread healthcare use.

## Conclusions

In conclusion, this study highlights the utility of ChatGPT as an adjunct in medical education and initial diagnostic assessments. While it excels in generating responses to structured queries, its performance in complex clinical scenarios requiring nuanced judgment is limited. The challenges in keeping the model up-to-date with the latest medical guidelines emphasize the need for continuous refinement and vigilant oversight. Healthcare professionals must remain central to the diagnostic process to ensure that digital tools augment, rather than supplant, their expertise. Future research should aim to improve the model’s understanding and application in clinical settings, ensuring its ethical and effective integration into healthcare practices.

## Electronic supplementary material

Below is the link to the electronic supplementary material.

Supplementary Material 1

Supplementary Material 2

Supplementary Material 3

## Data Availability

All data are avaliable as supplemental material.
