# Supplementary material for: Assessing ChatGPT’s theoretical knowledge and prescriptive accuracy in bacterial infections: a comparative study with infectious diseases residents and specialists
Source: Infection. 2024 Jul 12;53(3):873–81. doi: 10.1007/s15010-024-02350-6 (PMC12137519; doi:10.1007/s15010-024-02350-6)
Supplement: Supplementary file 1 — Supplementary Material 1 [file 15010_2024_2350_MOESM1_ESM.docx]

Table S1. Number of correct and wrong answers for the true or false questions divided by the themes; *p<0.001*

| True/False | Endocarditis | BSI | Pneumonia | Abdominal infection | Total |
| --- | --- | --- | --- | --- | --- |
| False | 16 (26.7) | 19 (31.7) | 30 (50.0) | 7 (11.7) | 72 (30.0) |
| True | 44 (73.3) | 41 (68.3) | 30 (50.0) | 53 (88.3) | 168 (70.0) |

Table S2. Number of correct and wrong answers for the true or false questions divided by the difficulty; *p*=0.009

| Accuracy | Easy | Medium | Hard | Total |
| --- | --- | --- | --- | --- |
| False | 21 (26.2) | 17 (21.2) | 34 (42.5) | 72 (30.0) |
| True | 59 (73.8) | 63 (78.2) | 46 (57.5) | 168 (70.0) |

Figure S1. Percentage of correct and wrong answers for the true or false questions in the different groups divided by the themes.


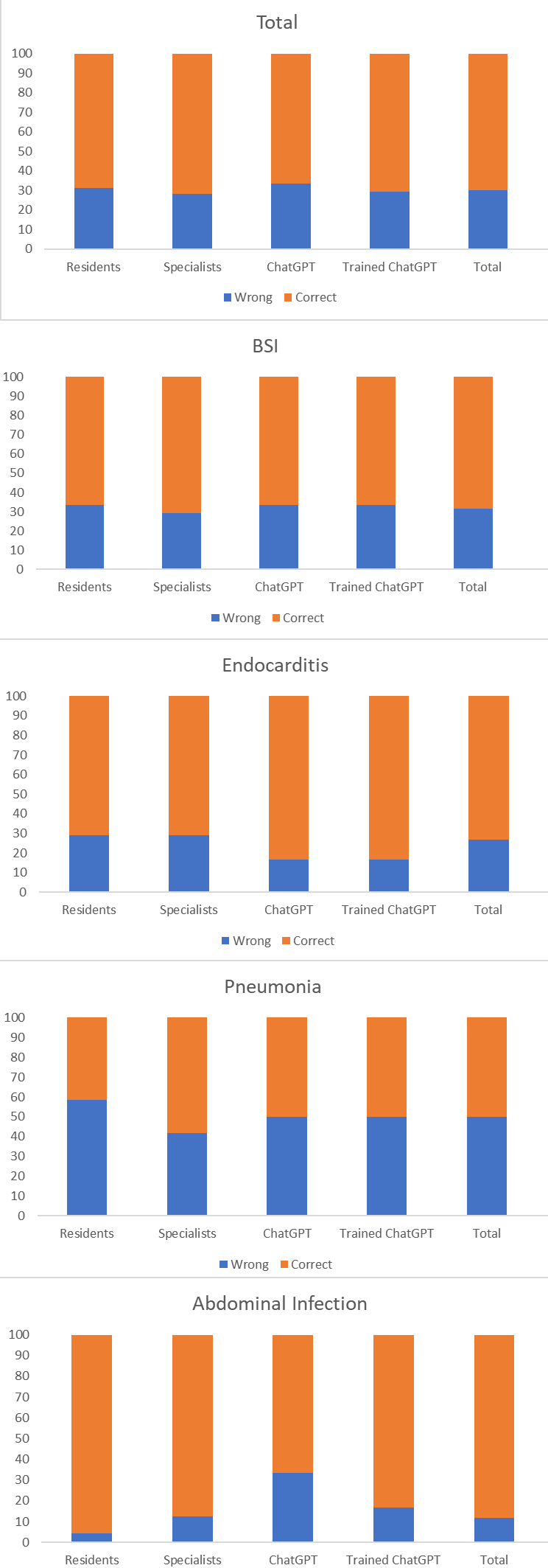


Table S3. Accuracy scoring of Infectious Diseases residents, specialists, ChatGPT4, and trained ChatGPT4 in answering open-ended antibiotic questions divided by the difficulty; *p*-value = 0.244

| Accuracy | Easy | Medium | Hard | Total |
| --- | --- | --- | --- | --- |
| 1 | 1 (1.2) | 3 (3.7) | 4 (5.0) | 8 (3.4) |
| 2 | 6 (7.5) | 9 (11.3) | 2 (2.5) | 17 (7.1) |
| 3 | 10 (12.5) | 17 (21.3) | 17 (21.3) | 44 (18.3) |
| 4 | 26 (32.5) | 22 (27.5) | 23 (28.7) | 71 (29.6) |
| 5 | 26 (32.5) | 23 (28.7) | 20 (25.0) | 69 (28.7) |
| 6 | 11 (13.8) | 6 (7.5) | 14 (17.5) | 31 (12.9) |

Table S4. Completeness scoring of Infectious Diseases residents, specialists, ChatGPT4, and trained ChatGPT4 in answering open-ended antibiotic questions divided by the difficulty; *p*-value = 0.264

| Completeness | Easy | Medium | Hard | Total |
| --- | --- | --- | --- | --- |
| 1 | 12 (15.0) | 23 (28.7) | 16 (20.0) | 51 (21.2) |
| 2 | 47 (58.8) | 43 (53.7) | 46 (57.5) | 136 (56.7) |
| 3 | 21 (26.2) | 14 (17.6) | 18 (22.5) | 53 (22.1) |

Table S5. Accuracy scoring of Infectious Diseases residents, specialists, ChatGPT4, and trained ChatGPT4 in answering open-ended antibiotic questions divided by the themes; *p*-value <0.001

| Accuracy | Endocarditis | BSI | Pneumonia | Abdominal infection | Total |
| --- | --- | --- | --- | --- | --- |
| 1 | 1 (1.7) | 0 | 4 (6.7) | 3 (5.0) | 8 (3.4) |
| 2 | 2 (3.4) | 4 (6.7) | 5 (8.3) | 6 (10.0) | 17 (7.1) |
| 3 | 4 (6.7) | 12 (20.0) | 13 (21.7) | 15 (25.0) | 44 (18.3) |
| 4 | 13 (21.7) | 24 (40.0) | 20 (33.3) | 14 (23.3) | 71 (29.6) |
| 5 | 17 (28.3) | 15 (25.0) | 17 (28.3 | 20 (33.3) | 69 (28.7) |
| 6 | 23 (38.2) | 5 (8.3) | 1 (1.7) | 2 (3.4) | 31 (12.9) |

Table S6. Completeness scoring of Infectious Diseases residents, specialists, ChatGPT4, and trained ChatGPT4 in answering open-ended antibiotic questions divided by the themes; *p-*value = *0.002*

| Completeness | Endocarditis | BSI | Pneumonia | Abdominal infection | Total |
| --- | --- | --- | --- | --- | --- |
| 1 | 14 (23.3) | 8 (13.3) | 17 (28.3) | 12 (20.0) | 51 (21.2) |
| 2 | 40 (66.7) | 28 (46.7) | 29 (48.3) | 39 (65.0) | 136 (56.7) |
| 3 | 6 (10.0) | 24 (40.0) | 14 (23.4) | 9 (15.0) | 53 (22.1) |

Table S7 Performance in identifying the correct resistance mechanisms according to the antibiogram divided by the question difficulty; *p-value* < 0.001

| Mechanism of resistance | Easy | Medium | Hard | Total |
| --- | --- | --- | --- | --- |
| Wrong | 18 (22.6) | 15 (18.7) | 16 (20.0) | 49 (20.4) |
| Partially Correct | 31 (38.7) | 44 (55.0) | 56 (70.0) | 131 (54.6) |
| Correct | 31 (38.7) | 21 (26.3) | 8 (10.0) | 60 (25.0) |

Table S8. Performance in prescribing the correct antibiotic treatment according to the clinical cases and the antibiograms divided by the question difficulty; *p*-value < 0.001

| Antibiotic choice | Easy | Medium | Hard | Total |
| --- | --- | --- | --- | --- |
| Wrong | 0 | 4 (5) | 18 (22.5) | 22 (9.2) |
| Partially Correct | 11 (13.7) | 13 (16.2) | 23 (28.7) | 47 (19.6) |
| Correct | 35 (43.8) | 37 (46.3) | 29 (36.3) | 101 (42.1) |
| Overtreatment | 34 (42.5) | 26 (32.5) | 10 (12.5) | 70 (29.1) |

Table S9. Performance in prescribing the correct length of treatment according to the clinical cases and the antibiograms divided by the question difficulty; *p*-value = 0.692

| Length of treatment | Easy | Medium | Hard | Total |
| --- | --- | --- | --- | --- |
| Short | 12 (15.0) | 11 (13.8) | 11 (13.8) | 34 (14.2) |
| Correct | 43 (53.8) | 51 (63.8) | 50 (62.5) | 144 (60.0) |
| Long | 25 (31.2) | 18 (22.5) | 19 (23.8) | 62 (25.8) |

Table S10. Performance in identifying the correct resistance mechanisms according to the antibiogram divided by the question themes; *p-value* = 0.004

| Mechanism of resistance | Endocarditis | BSI | Pneumonia | Abdominal infection | Total |
| --- | --- | --- | --- | --- | --- |
| Wrong | 7 (11.7) | 15 (25.0) | 16 (26.7) | 11 (18.3) | 49 (20.4) |
| Partially Correct | 38 (63.3) | 35 (58.3) | 20 (33.3) | 37 (63.3) | 131 (54.6) |
| Correct | 15 (25.0) | 10 (16.7) | 24 (40.0) | 11 (18.3) | 60 (25.0) |

Table S11. Performance in prescribing the correct antibiotic treatment according to the clinical cases and the antibiograms divided by the question theme; *p*-value = 0.004

| Antibiotic choice | Endocarditis | BSI | Pneumonia | Abdominal infection | Total |
| --- | --- | --- | --- | --- | --- |
| Wrong | 6 (10.0) | 8 (13.3) | 2 (3.3) | 6 (10.0) | 22 (9.2) |
| Partially Correct | 11 (18.3) | 12 (20.0) | 10 (16.7) | 14 (23.3) | 47 (19.6) |
| Correct | 24 (40.0) | 34 (56.7) | 19 (31.7) | 24 (40.0) | 101 (42.1) |
| Overtreatment | 19 (31.7) | 6 (10.0) | 29 (48.3) | 16 (26.7) | 70 (29.1) |

Table S12. Performance in prescribing the correct length of treatment according to the clinical cases and the antibiograms divided by the question theme; *p*-value = 0.001

| Length of treatment | Endocarditis | BSI | Pneumonia | Abdominal infection | Total |
| --- | --- | --- | --- | --- | --- |
| Short | 6 (10.0) | 14 (23.3) | 10 (16.7) | 4 (6.7) | 34 (14.2) |
| Correct | 44 (73.3) | 37 (61.7) | 34 (56.7) | 29 (48.3) | 144 (60.0) |
| Long | 10 (16.7) | 9 (15.0) | 16 (26.6) | 27 (45.0) | 62 (25.8) |
