# Supplementary material for: Assessing ChatGPT’s theoretical knowledge and prescriptive accuracy in bacterial infections: a comparative study with infectious diseases residents and specialists
Source: Infection. 2024 Jul 12;53(3):873–81. doi: 10.1007/s15010-024-02350-6 (PMC12137519; doi:10.1007/s15010-024-02350-6)
Supplement: Supplementary file 2 — Supplementary Material 2 [file 15010_2024_2350_MOESM2_ESM.docx]

1. Aldred, K. J., Kerns, R. J., & Osheroff, N. (2014). Mechanism of quinolone action and resistance. Biochemistry, 53(10), 1565–1574. <https://doi.org/10.1021/bi5000564>
2. Babich T, Naucler P, Valik JK, Giske CG, Benito N, Cardona R, Rivera A, Pulcini C, Fattah MA, Haquin J, Macgowan A, Grier S, Chazan B, Yanovskay A, Ami RB, Landes M, Nesher L, Zaidman-Shimshovitz A, McCarthy K, Paterson DL, Tacconelli E, Buhl M, Mauer S, Rodríguez-Baño J, de Cueto M, Oliver A, de Gopegui ER, Cano A, Machuca I, Gozalo-Marguello M, Martinez-Martinez L, Gonzalez-Barbera EM, Alfaro IG, Salavert M, Beovic B, Saje A, Mueller-Premru M, Pagani L, Vitrat V, Kofteridis D, Zacharioudaki M, Maraki S, Weissman Y, Paul M, Dickstein Y, Leibovici L, Yahav D. Duration of Treatment for Pseudomonas aeruginosa Bacteremia: a Retrospective Study. Infect Dis Ther. 2022 Aug;11(4):1505-1519. doi: 10.1007/s40121-022-00657-1. Epub 2022 May 25. PMID: 35612693; PMCID: PMC9334465.
3. Basatian-Tashkan B, Niakan M, Khaledi M, Afkhami H, Sameni F, Bakhti S, Mirnejad R. Antibiotic resistance assessment of Acinetobacter baumannii isolates from Tehran hospitals due to the presence of efflux pumps encoding genes (adeA and adeS genes) by molecular method. BMC Res Notes. 2020 Nov 19;13(1):543. doi: 10.1186/s13104-020-05387-6. PMID: 33213526; PMCID: PMC7678095.
4. Baseri, Z., Dehghan, A., Yaghoubi, S., & Razavi, S. (2021). Prevalence of resistance genes and antibiotic resistance profile among Stenotrophomonas maltophilia isolates from hospitalized patients in Iran. New microbes and new infections, 44, 100943. <https://doi.org/10.1016/j.nmni.2021.100943>
5. Bassetti M, Echols R, Matsunaga Y, Ariyasu M, Doi Y, Ferrer R, Lodise TP, Naas T, Niki Y, Paterson DL, Portsmouth S, Torre-Cisneros J, Toyoizumi K, Wunderink RG, Nagata TD. Efficacy and safety of cefiderocol or best available therapy for the treatment of serious infections caused by carbapenem-resistant Gram-negative bacteria (CREDIBLE-CR): a randomised, open-label, multicentre, pathogen-focused, descriptive, phase 3 trial. Lancet Infect Dis. 2021 Feb;21(2):226-240. doi: 10.1016/S1473-3099(20)30796-9. Epub 2020 Oct 12. PMID: 33058795.
6. Bergas A, Albasanz-Puig A, Fernández-Cruz A, Machado M, Novo A, van Duin D, Garcia-Vidal C, Hakki M, Ruiz-Camps I, Del Pozo JL, Oltolini C, DeVoe C, Drgona L, Gasch O, Mikulska M, Martín-Dávila P, Peghin M, Vázquez L, Laporte-Amargós J, Durà-Miralles X, Pallarès N, González-Barca E, Álvarez-Uría A, Puerta-Alcalde P, Aguilar-Company J, Carmona-Torre F, Clerici TD, Doernberg SB, Petrikova L, Capilla S, Magnasco L, Fortún J, Castaldo N, Carratalà J, Gudiol C. Real-Life Use of Ceftolozane/Tazobactam for the Treatment of Bloodstream Infection Due to Pseudomonas aeruginosa in Neutropenic Hematologic Patients: a Matched Control Study (ZENITH Study). Microbiol Spectr. 2022 Jun 29;10(3):e0229221. doi: 10.1128/spectrum.02292-21. Epub 2022 Apr 27. PMID: 35475683; PMCID: PMC9241913.
7. Biagi M, Vialichka A, Jurkovic M, Wu T, Shajee A, Lee M, Patel S, Mendes RE, Wenzler E. Activity of Cefiderocol Alone and in Combination with Levofloxacin, Minocycline, Polymyxin B, or Trimethoprim-Sulfamethoxazole against Multidrug-Resistant Stenotrophomonas maltophilia. Antimicrob Agents Chemother. 2020 Aug 20;64(9):e00559-20. doi: 10.1128/AAC.00559-20. PMID:
8. Boulekbache A, Maldonado F, Kavafian R, Ferry T, Bourguignon L, Goutelle S, Lega JC, Garreau R. Comparison of daptomycin and glycopeptide efficacy and safety for the treatment of Gram-positive infections: a systematic review and meta-analysis. J Antimicrob Chemother. 2024 Apr 2;79(4):712-721. doi: 10.1093/jac/dkae026. PMID: 38323372.
9. Bubp, J., Jen, M., & Matuszewski, K. (2015). Caring for Glucose-6-Phosphate Dehydrogenase (G6PD)-Deficient Patients: Implications for Pharmacy. P & T : a peer-reviewed journal for formulary management, 40(9), 572–574.
10. Carmeli Y, Armstrong J, Laud PJ, Newell P, Stone G, Wardman A, Gasink LB. Ceftazidime-avibactam or best available therapy in patients with ceftazidime-resistant Enterobacteriaceae and Pseudomonas aeruginosa complicated urinary tract infections or complicated intra-abdominal infections (REPRISE): a randomised, pathogen-directed, phase 3 study. Lancet Infect Dis. 2016 Jun;16(6):661-673. doi: 10.1016/S1473-3099(16)30004-4. Epub 2016 Apr 20. PMID: 27107460.
11. Cars O, Odenholt-Tornqvist I. The post-antibiotic sub-MIC effect in vitro and in vivo. J Antimicrob Chemother. 1993 May;31 Suppl D:159-66. doi: 10.1093/jac/31.suppl_d.159. PMID: 8335517.
12. Chaftari AM, Hachem R, Malek AE, Mulanovich VE, Szvalb AD, Jiang Y, Yuan Y, Ali S, Deeba R, Chaftari P, Raad I. A Prospective Randomized Study Comparing Ceftolozane/Tazobactam to Standard of Care in the Management of Neutropenia and Fever in Patients With Hematological Malignancies. Open Forum Infect Dis. 2022 Feb 14;9(6):ofac079. doi: 10.1093/ofid/ofac079. PMID: 35663286; PMCID: PMC9154317.
13. Chaves F, Garnacho-Montero J, Del Pozo JL, Bouza E, Capdevila JA, de Cueto M, Domínguez MÁ, Esteban J, Fernández-Hidalgo N, Fernández Sampedro M, Fortún J, Guembe M, Lorente L, Paño JR, Ramírez P, Salavert M, Sánchez M, Vallés J. Diagnosis and treatment of catheter-related bloodstream infection: Clinical guidelines of the Spanish Society of Infectious Diseases and Clinical Microbiology and (SEIMC) and the Spanish Society of Spanish Society of Intensive and Critical Care Medicine and Coronary Units (SEMICYUC). Med Intensiva (Engl Ed). 2018 Jan-Feb;42(1):5-36. English, Spanish. doi: 10.1016/j.medin.2017.09.012. PMID: 29406956.
14. Chen, H., Li, L., Liu, Y., Wu, M., Xu, S., Zhang, G., Qi, C., Du, Y., Wang, M., Li, J., & Huang, X. (2018). In vitro activity and post-antibiotic effects of linezolid in combination with fosfomycin against clinical isolates of Staphylococcus aureus. Infection and drug resistance, 11, 2107–2115. <https://doi.org/10.2147/IDR.S175978>
15. Clerici D, Oltolini C, Greco R, Ripa M, Giglio F, Mastaglio S, Lorentino F, Pavesi F, Farina F, Liberatore C, Castiglion B, Tassan Din C, Bernardi M, Corti C, Peccatori J, Scarpellini P, Ciceri F, Castagna A. The place of ceftazidime/avibactam and ceftolozane/tazobactam for therapy of haematological patients with febrile neutropenia. Int J Antimicrob Agents. 2021 Jun;57(6):106335. doi: 10.1016/j.ijantimicag.2021.106335. Epub 2021 Apr 7. PMID: 33838223.
16. Connell, S. R., Tracz, D. M., Nierhaus, K. H., & Taylor, D. E. (2003). Ribosomal protection proteins and their mechanism of tetracycline resistance. Antimicrobial agents and chemotherapy, 47(12), 3675–3681. <https://doi.org/10.1128/AAC.47.12.3675-3681.2003>
17. Delgado V, Ajmone Marsan N, de Waha S, Bonaros N, Brida M, Burri H, Caselli S, Doenst T, Ederhy S, Erba PA, Foldager D, Fosbøl EL, Kovac J, Mestres CA, Miller OI, Miro JM, Pazdernik M, Pizzi MN, Quintana E, Rasmussen TB, Ristić AD, Rodés-Cabau J, Sionis A, Zühlke LJ, Borger MA; ESC Scientific Document Group. 2023 ESC Guidelines for the management of endocarditis. Eur Heart J. 2023 Oct 14;44(39):3948-4042. doi: 10.1093/eurheartj/ehad193. Erratum in: Eur Heart J. 2023 Sep 20;: Erratum in: Eur Heart J. 2024 Jan 1;45(1):56. PMID: 37622656.
18. Evans L, Rhodes A, Alhazzani W, Antonelli M, Coopersmith CM, French C, Machado FR, Mcintyre L, Ostermann M, Prescott HC, Schorr C, Simpson S, Wiersinga WJ, Alshamsi F, Angus DC, Arabi Y, Azevedo L, Beale R, Beilman G, Belley-Cote E, Burry L, Cecconi M, Centofanti J, Coz Yataco A, De Waele J, Dellinger RP, Doi K, Du B, Estenssoro E, Ferrer R, Gomersall C, Hodgson C, Møller MH, Iwashyna T, Jacob S, Kleinpell R, Klompas M, Koh Y, Kumar A, Kwizera A, Lobo S, Masur H, McGloughlin S, Mehta S, Mehta Y, Mer M, Nunnally M, Oczkowski S, Osborn T, Papathanassoglou E, Perner A, Puskarich M, Roberts J, Schweickert W, Seckel M, Sevransky J, Sprung CL, Welte T, Zimmerman J, Levy M. Surviving sepsis campaign: international guidelines for management of sepsis and septic shock 2021. Intensive Care Med. 2021 Nov;47(11):1181-1247. doi: 10.1007/s00134-021-06506-y. Epub 2021 Oct 2. PMID: 34599691; PMCID: PMC8486643.
19. Falcone M, Tiseo G, Leonildi A, Della Sala L, Vecchione A, Barnini S, Farcomeni A, Menichetti F. Cefiderocol- Compared to Colistin-Based Regimens for the Treatment of Severe Infections Caused by Carbapenem-Resistant Acinetobacter baumannii. Antimicrob Agents Chemother. 2022 May 17;66(5):e0214221. doi: 10.1128/aac.02142-21. Epub 2022 Mar 21. PMID: 35311522; PMCID: PMC9112922.
20. Foster TJ. Antibiotic resistance in Staphylococcus aureus. Current status and future prospects. FEMS Microbiol Rev. 2017 May 1;41(3):430-449. doi: 10.1093/femsre/fux007. PMID: 28419231.
21. Frank JE. Diagnosis and management of G6PD deficiency. Am Fam Physician. 2005 Oct 1;72(7):1277-82. PMID: 16225031.
22. Georgina Solano-Gálvez, S., Fernanda Valencia-Segrove, M., José Ostos Prado, M., Berenice López Boucieguez, A., Abelardo Álvarez-Hernández, D., & Vázquez-López, R. (2021). Mechanisms of Resistance to Quinolones. IntechOpen. doi: 10.5772/intechopen.92577
23. Gibb, J., & Wong, D. W. (2021). Antimicrobial Treatment Strategies for Stenotrophomonas maltophilia: A Focus on Novel Therapies. Antibiotics (Basel, Switzerland), 10(10), 1226. <https://doi.org/10.3390/antibiotics10101226>
24. Giovagnorio F, De Vito A, Madeddu G, Parisi SG, Geremia N. Resistance in *Pseudomonas aeruginosa*: A Narrative Review of Antibiogram Interpretation and Emerging Treatments. Antibiotics (Basel). 2023 Nov 12;12(11):1621. doi: 10.3390/antibiotics12111621. PMID: 37998823; PMCID: PMC10669487.
25. Grossman TH. Tetracycline Antibiotics and Resistance. Cold Spring Harb Perspect Med. 2016;6(4):a025387. Published 2016 Apr 1. doi:10.1101/cshperspect.a025387
26. Gu B, Kelesidis T, Tsiodras S, Hindler J, Humphries RM. The emerging problem of linezolid-resistant Staphylococcus. J Antimicrob Chemother. 2013 Jan;68(1):4-11. doi: 10.1093/jac/dks354. Epub 2012 Sep 4. PMID: 22949625; PMCID: PMC8445637.
27. Guo Y, Song G, Sun M, Wang J, Wang Y. Prevalence and Therapies of Antibiotic-Resistance in Staphylococcus aureus. Front Cell Infect Microbiol. 2020;10:107. Published 2020 Mar 17. doi:10.3389/fcimb.2020.00107
28. Harris PNA, Tambyah PA, Lye DC, Mo Y, Lee TH, Yilmaz M, Alenazi TH, Arabi Y, Falcone M, Bassetti M, Righi E, Rogers BA, Kanj S, Bhally H, Iredell J, Mendelson M, Boyles TH, Looke D, Miyakis S, Walls G, Al Khamis M, Zikri A, Crowe A, Ingram P, Daneman N, Griffin P, Athan E, Lorenc P, Baker P, Roberts L, Beatson SA, Peleg AY, Harris-Brown T, Paterson DL; MERINO Trial Investigators and the Australasian Society for Infectious Disease Clinical Research Network (ASID-CRN). Effect of Piperacillin-Tazobactam vs Meropenem on 30-Day Mortality for Patients With E coli or Klebsiella pneumoniae Bloodstream Infection and Ceftriaxone Resistance: A Randomized Clinical Trial. JAMA. 2018 Sep 11;320(10):984-994. doi: 10.1001/jama.2018.12163. Erratum in: JAMA. 2019 Jun 18;321(23):2370. PMID: 30208454; PMCID: PMC6143100.
29. Holland TL, Cosgrove SE, Doernberg SB, Jenkins TC, Turner NA, Boucher HW, Pavlov O, Titov I, Kosulnykov S, Atanasov B, Poromanski I, Makhviladze M, Anderzhanova A, Stryjewski ME, Assadi Gehr M, Engelhardt M, Hamed K, Ionescu D, Jones M, Saulay M, Smart J, Seifert H, Fowler VG Jr; ERADICATE Study Group. Ceftobiprole for Treatment of Complicated *Staphylococcus aureus* Bacteremia. N Engl J Med. 2023 Oct 12;389(15):1390-1401. doi: 10.1056/NEJMoa2300220. Epub 2023 Sep 27. PMID: 37754204.
30. Hong LT, Downes KJ, FakhriRavari A, Abdul-Mutakabbir JC, Kuti JL, Jorgensen S, Young DC, Alshaer MH, Bassetti M, Bonomo RA, Gilchrist M, Jang SM, Lodise T, Roberts JA, Tängdén T, Zuppa A, Scheetz MH. International consensus recommendations for the use of prolonged-infusion beta-lactam antibiotics: Endorsed by the American College of Clinical Pharmacy, British Society for Antimicrobial Chemotherapy, Cystic Fibrosis Foundation, European Society of Clinical Microbiology and Infectious Diseases, Infectious Diseases Society of America, Society of Critical Care Medicine, and Society of Infectious Diseases Pharmacists. Pharmacotherapy. 2023 Aug;43(8):740-777. doi: 10.1002/phar.2842. Erratum in: Pharmacotherapy. 2023 Sep 19;: Erratum in: Pharmacotherapy. 2024 Jan 11;: PMID: 37615245.
31. Hooper, D. C., & Jacoby, G. A. (2015). Mechanisms of drug resistance: quinolone resistance. Annals of the New York Academy of Sciences, 1354(1), 12–31. <https://doi.org/10.1111/nyas.12830>
32. Huovinen P. Resistance to trimethoprim-sulfamethoxazole. Clin Infect Dis. 2001 Jun 1;32(11):1608-14. doi: 10.1086/320532. Epub 2001 May 4. PMID: 11340533.
33. Isler B, Kidd TJ, Stewart AG, Harris P, Paterson DL. *Achromobacter* Infections and Treatment Options. Antimicrob Agents Chemother. 2020 Oct 20;64(11):e01025-20. doi: 10.1128/AAC.01025-20. PMID: 32816734; PMCID: PMC7577122.
34. Jackson CA, Newland J, Dementieva N, Lonchar J, Su FH, Huntington JA, Bensaci M, Popejoy MW, Johnson MG, De Anda C, Rhee EG, Bruno CJ. Safety and Efficacy of Ceftolozane/Tazobactam Plus Metronidazole Versus Meropenem From a Phase 2, Randomized Clinical Trial in Pediatric Participants With Complicated Intra-abdominal Infection. Pediatr Infect Dis J. 2023 Jul 1;42(7):557-563. doi: 10.1097/INF.0000000000003911. Epub 2023 Mar 29. PMID: 37000942; PMCID: PMC10259210.
35. Jame W, Basgut B, Abdi A. Efficacy and safety of novel glycopeptides versus vancomycin for the treatment of gram-positive bacterial infections including methicillin resistant Staphylococcus aureus: A systematic review and meta-analysis. PLoS One. 2021 Nov 29;16(11):e0260539. doi: 10.1371/journal.pone.0260539. PMID: 34843561; PMCID: PMC8629313.
36. Justo JA, Bookstaver PB. Antibiotic lock therapy: review of technique and logistical challenges. Infect Drug Resist. 2014 Dec 12;7:343-63. doi: 10.2147/IDR.S51388. PMID: 25548523; PMCID: PMC4271721.
37. Kaye KS, Bhowmick T, Metallidis S, Bleasdale SC, Sagan OS, Stus V, Vazquez J, Zaitsev V, Bidair M, Chorvat E, Dragoescu PO, Fedosiuk E, Horcajada JP, Murta C, Sarychev Y, Stoev V, Morgan E, Fusaro K, Griffith D, Lomovskaya O, Alexander EL, Loutit J, Dudley MN, Giamarellos-Bourboulis EJ. Effect of Meropenem-Vaborbactam vs Piperacillin-Tazobactam on Clinical Cure or Improvement and Microbial Eradication in Complicated Urinary Tract Infection: The TANGO I Randomized Clinical Trial. JAMA. 2018 Feb 27;319(8):788-799. doi: 10.1001/jama.2018.0438. PMID: 29486041; PMCID: PMC5838656.
38. Kern WV, Oethinger M, Kaufhold A, Rozdzinski E, Marre R. Ochrobactrum anthropi bacteremia: report of four cases and short review. Infection. 1993 Sep-Oct;21(5):306-10. doi: 10.1007/BF01712451. PMID: 8300247.
39. Kidd TJ, Mills G, Sá-Pessoa J, Dumigan A, Frank CG, Insua JL, Ingram R, Hobley L, Bengoechea JA. A *Klebsiella pneumoniae* antibiotic resistance mechanism that subdues host defences and promotes virulence. EMBO Mol Med. 2017 Apr;9(4):430-447. doi: 10.15252/emmm.201607336. PMID: 28202493; PMCID: PMC5376759.
40. Kollef MH, Nováček M, Kivistik Ü, Réa-Neto Á, Shime N, Martin-Loeches I, Timsit JF, Wunderink RG, Bruno CJ, Huntington JA, Lin G, Yu B, Butterton JR, Rhee EG. Ceftolozane-tazobactam versus meropenem for treatment of nosocomial pneumonia (ASPECT-NP): a randomised, controlled, double-blind, phase 3, non-inferiority trial. Lancet Infect Dis. 2019 Dec;19(12):1299-1311. doi: 10.1016/S1473-3099(19)30403-7. Epub 2019 Sep 25. PMID: 31563344.
41. Kollef MH, Ricard JD, Roux D, Francois B, Ischaki E, Rozgonyi Z, Boulain T, Ivanyi Z, János G, Garot D, Koura F, Zakynthinos E, Dimopoulos G, Torres A, Danker W, Montgomery AB. A Randomized Trial of the Amikacin Fosfomycin Inhalation System for the Adjunctive Therapy of Gram-Negative Ventilator-Associated Pneumonia: IASIS Trial. Chest. 2017 Jun;151(6):1239-1246. doi: 10.1016/j.chest.2016.11.026. Epub 2016 Nov 24. PMID: 27890714.
42. Leclercq R. Mechanisms of resistance to macrolides and lincosamides: nature of the resistance elements and their clinical implications. Clin Infect Dis. 2002 Feb 15;34(4):482-92. doi: 10.1086/324626. Epub 2002 Jan 11. PMID: 11797175.
43. Li W, Atkinson GC, Thakor NS, Allas U, Lu CC, Chan KY, Tenson T, Schulten K, Wilson KS, Hauryliuk V, Frank J. Mechanism of tetracycline resistance by ribosomal protection protein Tet(O). Nat Commun. 2013;4:1477. doi: 10.1038/ncomms2470. PMID: 23403578; PMCID: PMC3576927.
44. Li Y, Kumar S, Zhang L, Wu H, Wu H. Characteristics of antibiotic resistance mechanisms and genes of *Klebsiella pneumoniae*. *Open Med (Wars)*. 2023;18(1):20230707. Published 2023 May 12. doi:10.1515/med-2023-0707
45. Long, K. S., & Vester, B. (2012). Resistance to linezolid caused by modifications at its binding site on the ribosome. Antimicrobial agents and chemotherapy, 56(2), 603–612. <https://doi.org/10.1128/AAC.05702-11>
46. Luque Paz D, Chean D, Tattevin P, Luque Paz D, Bayeh BA, Kouatchet A, Douillet D, Riou J. Efficacy and safety of antibiotics targeting Gram-negative bacteria in nosocomial pneumonia: a systematic review and Bayesian network meta-analysis. Ann Intensive Care. 2024 Apr 25;14(1):66. doi: 10.1186/s13613-024-01291-5. PMID: 38662091; PMCID: PMC11045692.
47. MacKenzie FM, Gould IM. The post-antibiotic effect. J Antimicrob Chemother. 1993 Oct;32(4):519-37. doi: 10.1093/jac/32.4.519. PMID: 8288494.
48. Manna MS, Tamer YT, Gaszek I, Poulides N, Ahmed A, Wang X, Toprak FCR, Woodard DR, Koh AY, Williams NS, Borek D, Atilgan AR, Hulleman JD, Atilgan C, Tambar U, Toprak E. A trimethoprim derivative impedes antibiotic resistance evolution. Nat Commun. 2021 May 19;12(1):2949. doi: 10.1038/s41467-021-23191-z. PMID: 34011959; PMCID: PMC8134463.
49. Martinez-Nadal G, Puerta-Alcalde P, Gudiol C, Cardozo C, Albasanz-Puig A, Marco F, Laporte-Amargós J, Moreno-García E, Domingo-Doménech E, Chumbita M, Martínez JA, Soriano A, Carratalà J, Garcia-Vidal C. Inappropriate Empirical Antibiotic Treatment in High-risk Neutropenic Patients With Bacteremia in the Era of Multidrug Resistance. Clin Infect Dis. 2020 Mar 3;70(6):1068-1074. doi: 10.1093/cid/ciz319. PMID: 31321410.
50. Martin-Loeches I, Torres A, Nagavci B, Aliberti S, Antonelli M, Bassetti M, Bos LD, Chalmers JD, Derde L, de Waele J, Garnacho-Montero J, Kollef M, Luna CM, Menendez R, Niederman MS, Ponomarev D, Restrepo MI, Rigau D, Schultz MJ, Weiss E, Welte T, Wunderink R. ERS/ESICM/ESCMID/ALAT guidelines for the management of severe community-acquired pneumonia. Intensive Care Med. 2023 Jun;49(6):615-632. doi: 10.1007/s00134-023-07033-8. Epub 2023 Apr 4. Erratum in: Intensive Care Med. 2023 May 17;: PMID: 37012484; PMCID: PMC10069946.
51. Mastroianni A, Cancellieri C, Montini G. Ochrobactrum anthropi bacteremia: case report and review of the literature. Clin Microbiol Infect. 1999 Sep;5(9):570-573. doi: 10.1111/j.1469-0691.1999.tb00437.x. PMID: 11851711.
52. Metlay JP, Waterer GW, Long AC, Anzueto A, Brozek J, Crothers K, Cooley LA, Dean NC, Fine MJ, Flanders SA, Griffin MR, Metersky ML, Musher DM, Restrepo MI, Whitney CG. Diagnosis and Treatment of Adults with Community-acquired Pneumonia. An Official Clinical Practice Guideline of the American Thoracic Society and Infectious Diseases Society of America. Am J Respir Crit Care Med. 2019 Oct 1;200(7):e45-e67. doi: 10.1164/rccm.201908-1581ST. PMID: 31573350; PMCID: PMC6812437.
53. Mlynarczyk-Bonikowska B, Kowalewski C, Krolak-Ulinska A, Marusza W. Molecular Mechanisms of Drug Resistance in *Staphylococcus aureus*. *Int J Mol Sci*. 2022;23(15):8088. Published 2022 Jul 22. doi:10.3390/ijms23158088
54. Mojica MF, Humphries R, Lipuma JJ, Mathers AJ, Rao GG, Shelburne SA, Fouts DE, Van Duin D, Bonomo RA. Clinical challenges treating *Stenotrophomonas maltophilia* infections: an update. JAC Antimicrob Resist. 2022 May 5;4(3):dlac040. doi: 10.1093/jacamr/dlac040. PMID: 35529051; PMCID: PMC9071536.
55. Motsch J, Murta de Oliveira C, Stus V, Köksal I, Lyulko O, Boucher HW, Kaye KS, File TM, Brown ML, Khan I, Du J, Joeng HK, Tipping RW, Aggrey A, Young K, Kartsonis NA, Butterton JR, Paschke A. RESTORE-IMI 1: A Multicenter, Randomized, Double-blind Trial Comparing Efficacy and Safety of Imipenem/Relebactam vs Colistin Plus Imipenem in Patients With Imipenem-nonsusceptible Bacterial Infections. Clin Infect Dis. 2020 Apr 15;70(9):1799-1808. doi: 10.1093/cid/ciz530. PMID: 31400759; PMCID: PMC7156774.
56. Norris LB, Kablaoui F, Brilhart MK, Bookstaver PB. Systematic review of antimicrobial lock therapy for prevention of central-line-associated bloodstream infections in adult and pediatric cancer patients. Int J Antimicrob Agents. 2017 Sep;50(3):308-317. doi: 10.1016/j.ijantimicag.2017.06.013. Epub 2017 Jul 6. PMID: 28689878.
57. O'Grady, N. P., Alexander, M., Burns, L. A., Dellinger, E. P., Garland, J., Heard, S. O., Lipsett, P. A., Masur, H., Mermel, L. A., Pearson, M. L., Raad, I. I., Randolph, A. G., Rupp, M. E., Saint, S., & Healthcare Infection Control Practices Advisory Committee (HICPAC) (Appendix 1) (2011). Summary of recommendations: Guidelines for the Prevention of Intravascular Catheter-related Infections. Clinical infectious diseases : an official publication of the Infectious Diseases Society of America, 52(9), 1087–1099. <https://doi.org/10.1093/cid/cir138>
58. Paterson DL, Kinoshita M, Baba T, Echols R, Portsmouth S. Outcomes with Cefiderocol Treatment in Patients with Bacteraemia Enrolled into Prospective Phase 2 and Phase 3 Randomised Clinical Studies. *Infect Dis Ther*. 2022;11(2):853-870. doi:10.1007/s40121-022-00598-9
59. Qian ET, Casey JD, Wright A, et al. Cefepime vs Piperacillin-Tazobactam in Adults Hospitalized With Acute Infection: The ACORN Randomized Clinical Trial. JAMA. 2023;330(16):1557–1567. doi:10.1001/jama.2023.20583
60. Ramos JM, Román A, Fernández-Roblas R, Cabello A, Soriano F. Infection caused by Ochrobactrum anthropi. Clin Microbiol Infect. 1996 Mar;1(3):214-216. doi: 10.1111/j.1469-0691.1996.tb00561.x. PMID: 11866765.
61. Richardson SR, O'Malley GF. Glucose-6-Phosphate Dehydrogenase Deficiency. [Updated 2022 Sep 26]. In: StatPearls [Internet]. Treasure Island (FL): StatPearls Publishing; 2024 Jan-. Available from: <https://www.ncbi.nlm.nih.gov/books/NBK470315/>
62. Sartelli, M., Coccolini, F., Kluger, Y. *et al.* WSES/GAIS/SIS-E/WSIS/AAST global clinical pathways for patients with intra-abdominal infections. *World J Emerg Surg* **16**, 49 (2021). https://doi.org/10.1186/s13017-021-00387-8
63. Seifi N, Kahani N, Askari E, Mahdipour S, Naderi NM. Inducible clindamycin resistance in Staphylococcus aureus isolates recovered from Mashhad, Iran. Iran J Microbiol. 2012 Jun;4(2):82-6. PMID: 22973474; PMCID: PMC3434646.
64. Signorino, C., Fusco, E., Galli, L., & Chiappini, E. (2023). Effectiveness of Antimicrobial Lock Therapy for the Treatment of Catheter-Related and Central-Line-Associated Bloodstream Infections in Children: A Single Center Retrospective Study. Antibiotics (Basel, Switzerland), 12(5), 800. <https://doi.org/10.3390/antibiotics12050800>
65. Singh H, Thangaraj P, Chakrabarti A. Acinetobacter baumannii: A Brief Account of Mechanisms of Multidrug Resistance and Current and Future Therapeutic Management. J Clin Diagn Res. 2013 Nov;7(11):2602-5. doi: 10.7860/JCDR/2013/6337.3626. Epub 2013 Nov 10. PMID: 24392418; PMCID: PMC3879836.
66. Sojo-Dorado J, López-Hernández I, Rosso-Fernandez C, et al. Effectiveness of Fosfomycin for the Treatment of Multidrug-Resistant Escherichia coli Bacteremic Urinary Tract Infections: A Randomized Clinical Trial. *JAMA Netw Open*. 2022;5(1):e2137277. Published 2022 Jan 4. doi:10.1001/jamanetworkopen.2021.37277
67. Soto CL, Hsu AJ, Lee JH, Dzintars K, Choudhury R, Jenkins TC, McCreary EK, Quartuccio KS, Stohs EJ, Zimmerman M, Tamma PD. Identifying Effective Durations of Antibiotic Therapy for the Treatment of Carbapenem-resistant Enterobacterales Bloodstream Infections: A Multicenter Observational Study. Clin Infect Dis. 2024 Jan 25;78(1):27-30. doi: 10.1093/cid/ciad476. PMID: 37584360.
68. Spangler SK, Lin G, Jacobs MR, Appelbaum PC. Postantibiotic effect and postantibiotic sub-MIC effect of levofloxacin compared to those of ofloxacin, ciprofloxacin, erythromycin, azithromycin, and clarithromycin against 20 pneumococci. Antimicrob Agents Chemother. 1998 May;42(5):1253-5. doi: 10.1128/AAC.42.5.1253. PMID: 9593160; PMCID: PMC105793.
69. Srimani JK, Huang S, Lopatkin AJ, You L. Drug detoxification dynamics explain the postantibiotic effect. Mol Syst Biol. 2017 Oct 23;13(10):948. doi: 10.15252/msb.20177723. PMID: 29061668; PMCID: PMC5658699.
70. Stefani S, Bongiorno D, Mongelli G, Campanile F. Linezolid Resistance in Staphylococci. Pharmaceuticals. 2010; 3(7):1988-2006. https://doi.org/10.3390/ph3071988
71. Stewart AG, Paterson DL, Young B, Lye DC, Davis JS, Schneider K, Yilmaz M, Dinleyici R, Runnegar N, Henderson A, Archuleta S, Kalimuddin S, Forde BM, Chatfield MD, Bauer MJ, Lipman J, Harris-Brown T, Harris PNA; MERINO Trial Investigators and the Australasian Society for Infectious Disease Clinical Research Network (ASID-CRN). Meropenem Versus Piperacillin-Tazobactam for Definitive Treatment of Bloodstream Infections Caused by AmpC β-Lactamase-Producing *Enterobacter* spp, *Citrobacter freundii*, *Morganella morganii*, *Providencia* spp, or *Serratia marcescens*: A Pilot Multicenter Randomized Controlled Trial (MERINO-2). Open Forum Infect Dis. 2021 Aug 2;8(8):ofab387. doi: 10.1093/ofid/ofab387. PMID: 34395716; PMCID: PMC8361238.
72. Thabit AK. Antibiotics in the Biliary Tract: A Review of the Pharmacokinetics and Clinical Outcomes of Antibiotics Penetrating the Bile and Gallbladder Wall. Pharmacotherapy. 2020 Jul;40(7):672-691. doi: 10.1002/phar.2431. Epub 2020 Jun 25. PMID: 32485056.
73. Thoma B, Straube E, Scholz HC, Al Dahouk S, Zöller L, Pfeffer M, Neubauer H, Tomaso H. Identification and antimicrobial susceptibilities of Ochrobactrum spp. Int J Med Microbiol. 2009 Mar;299(3):209-20. doi: 10.1016/j.ijmm.2008.06.009. Epub 2008 Aug 27. PMID: 18755630.
74. Timsit JF, Baleine J, Bernard L, Calvino-Gunther S, Darmon M, Dellamonica J, Desruennes E, Leone M, Lepape A, Leroy O, Lucet JC, Merchaoui Z, Mimoz O, Misset B, Parienti JJ, Quenot JP, Roch A, Schmidt M, Slama M, Souweine B, Zahar JR, Zingg W, Bodet-Contentin L, Maxime V. Expert consensus-based clinical practice guidelines management of intravascular catheters in the intensive care unit. Ann Intensive Care. 2020 Sep 7;10(1):118. doi: 10.1186/s13613-020-00713-4. PMID: 32894389; PMCID: PMC7477021.
75. Tiseo G, Brigante G, Giacobbe DR, Maraolo AE, Gona F, Falcone M, Giannella M, Grossi P, Pea F, Rossolini GM, Sanguinetti M, Sarti M, Scarparo C, Tumbarello M, Venditti M, Viale P, Bassetti M, Luzzaro F, Menichetti F, Stefani S, Tinelli M. Diagnosis and management of infections caused by multidrug-resistant bacteria: guideline endorsed by the Italian Society of Infection and Tropical Diseases (SIMIT), the Italian Society of Anti-Infective Therapy (SITA), the Italian Group for Antimicrobial Stewardship (GISA), the Italian Association of Clinical Microbiologists (AMCLI) and the Italian Society of Microbiology (SIM). Int J Antimicrob Agents. 2022 Aug;60(2):106611. doi: 10.1016/j.ijantimicag.2022.106611. Epub 2022 Jun 11. PMID: 35697179.
76. Titov I, Wunderink RG, Roquilly A, Rodríguez Gonzalez D, David-Wang A, Boucher HW, Kaye KS, Losada MC, Du J, Tipping R, Rizk ML, Patel M, Brown ML, Young K, Kartsonis NA, Butterton JR, Paschke A, Chen LF. A Randomized, Double-blind, Multicenter Trial Comparing Efficacy and Safety of Imipenem/Cilastatin/Relebactam Versus Piperacillin/Tazobactam in Adults With Hospital-acquired or Ventilator-associated Bacterial Pneumonia (RESTORE-IMI 2 Study). Clin Infect Dis. 2021 Dec 6;73(11):e4539-e4548. doi: 10.1093/cid/ciaa803. PMID: 32785589; PMCID: PMC8662781.
77. Torres A, Wible M, Tawadrous M, Irani P, Stone GG, Quintana A, Debabov D, Burroughs M, Bradford PA, Kollef M. Efficacy and safety of ceftazidime/avibactam in patients with infections caused by β-lactamase-producing Gram-negative pathogens: a pooled analysis from the Phase 3 clinical trial programme. J Antimicrob Chemother. 2023 Nov 6;78(11):2672-2682. doi: 10.1093/jac/dkad280. PMID: 37700689.
78. Trecarichi EM, Tumbarello M, Caira M, Candoni A, Cattaneo C, Pastore D, Fanci R, Nosari A, Vianelli N, Busca A, Spadea A, Pagano L. Multidrug resistant Pseudomonas aeruginosa bloodstream infection in adult patients with hematologic malignancies. Haematologica. 2011 Jan;96(1):e1-3; author reply e4. doi: 10.3324/haematol.2010.036640. PMID: 21193424; PMCID: PMC3012771.
79. Turjeman A, von Dach E, Molina J, et al. Duration of antibiotic treatment for Gram-negative bacteremia - Systematic review and individual participant data (IPD) meta-analysis. *EClinicalMedicine*. 2022;55:101750. Published 2022 Dec 1. doi:10.1016/j.eclinm.2022.101750
80. Vardakas KZ, Mavros MN, Roussos N, Falagas ME. Meta-analysis of randomized controlled trials of vancomycin for the treatment of patients with gram-positive infections: focus on the study design. Mayo Clin Proc. 2012 Apr;87(4):349-63. doi: 10.1016/j.mayocp.2011.12.011. PMID: 22469348; PMCID: PMC3538415.
81. Vassallo M, Dunais B, Roger PM. Antimicrobial lock therapy in central-line associated bloodstream infections: a systematic review. Infection. 2015 Aug;43(4):389-98. doi: 10.1007/s15010-015-0738-1. Epub 2015 Feb 6. PMID: 25657033.
82. Wang Y, Sun X. Reevaluation of lock solutions for Central venous catheters in hemodialysis: a narrative review. Ren Fail. 2022 Dec;44(1):1501-1518. doi: 10.1080/0886022X.2022.2118068. PMID: 36047812; PMCID: PMC9448397.
83. Weinberg SE, Villedieu A, Bagdasarian N, Karah N, Teare L, Elamin WF. Control and management of multidrug resistant *Acinetobacter baumannii*: A review of the evidence and proposal of novel approaches. Infect Prev Pract. 2020 Jul 19;2(3):100077. doi: 10.1016/j.infpip.2020.100077. PMID: 34368717; PMCID: PMC8336160.
84. Wunderink RG, Giamarellos-Bourboulis EJ, Rahav G, Mathers AJ, Bassetti M, Vazquez J, Cornely OA, Solomkin J, Bhowmick T, Bishara J, Daikos GL, Felton T, Furst MJL, Kwak EJ, Menichetti F, Oren I, Alexander EL, Griffith D, Lomovskaya O, Loutit J, Zhang S, Dudley MN, Kaye KS. Effect and Safety of Meropenem-Vaborbactam versus Best-Available Therapy in Patients with Carbapenem-Resistant Enterobacteriaceae Infections: The TANGO II Randomized Clinical Trial. Infect Dis Ther. 2018 Dec;7(4):439-455. doi: 10.1007/s40121-018-0214-1. Epub 2018 Oct 1. PMID: 30270406; PMCID: PMC6249182.
85. Yang J, Zhang K, Ding C, Wang S, Wu W, Liu X. Exploring multidrug-resistant Klebsiella pneumoniae antimicrobial resistance mechanisms through whole genome sequencing analysis. BMC Microbiol. 2023 Sep 2;23(1):245. doi: 10.1186/s12866-023-02974-y. PMID: 37660028; PMCID: PMC10474722.
86. Yoon EJ, Jeong SH. Class D β-lactamases. J Antimicrob Chemother. 2021 Mar 12;76(4):836-864. doi: 10.1093/jac/dkaa513. PMID: 33382875.
87. Zhao X, Huang H, Yuan H, Yuan Z, Zhang Y. A Phase III multicentre, randomized, double-blind trial to evaluate the efficacy and safety of oral contezolid versus linezolid in adults with complicated skin and soft tissue infections. J Antimicrob Chemother. 2022 May 29;77(6):1762-1769. doi: 10.1093/jac/dkac073. PMID: 35265985.
88. Zhu M, Zhao X, Zhu Q, Zhang Z, Dai Y, Chen L, Liang Z. Clinical characteristics of patients with Ochrobactrum anthropi bloodstream infection in a Chinese tertiary-care hospital: A 7-year study. J Infect Public Health. 2018 Nov-Dec;11(6):873-877. doi: 10.1016/j.jiph.2018.07.009. Epub 2018 Aug 9. PMID: 30100242.
